# A Path to Implement Precision Child Health Cardiovascular Medicine

**DOI:** 10.3389/fcvm.2017.00036

**Published:** 2017-06-01

**Authors:** Marlin Touma, Brian Reemtsen, Nancy Halnon, Juan Alejos, J. Paul Finn, Stanley F. Nelson, Yibin Wang

**Affiliations:** ^1^Department of Pediatrics, Children’s Discovery and Innovation Institute, University of California at Los Angeles, Los Angeles, CA, United States; ^2^Cardiovascular Research Laboratory, University of California at Los Angeles, Los Angeles, CA, United States; ^3^Department of Cardiothoracic Surgery, University of California at Los Angeles, Los Angeles, CA, United States; ^4^Department of Pediatrics, University of California at Los Angeles, Los Angeles, CA, United States; ^5^Department of Radiology, Cardiovascular Imaging, University of California at Los Angeles, Los Angeles, CA, United States; ^6^Department of Human Genetics, University of California at Los Angeles, Los Angeles, CA, United States; ^7^Department of Anesthesiology, Physiology and Medicine, University of California at Los Angeles, Los Angeles, CA, United States

**Keywords:** congenital heart defects, bio banking, whole-exome sequencing, RNA-sequencing, transcriptome, variants, repository

## Abstract

Congenital heart defects (CHDs) affect approximately 1% of live births and are a major source of childhood morbidity and mortality even in countries with advanced healthcare systems. Along with phenotypic heterogeneity, the underlying etiology of CHDs is multifactorial, involving genetic, epigenetic, and/or environmental contributors. Clear dissection of the underlying mechanism is a powerful step to establish individualized therapies. However, the majority of CHDs are yet to be clearly diagnosed for the underlying genetic and environmental factors, and even less with effective therapies. Although the survival rate for CHDs is steadily improving, there is still a significant unmet need for refining diagnostic precision and establishing targeted therapies to optimize life quality and to minimize future complications. In particular, proper identification of disease associated genetic variants in humans has been challenging, and this greatly impedes our ability to delineate gene–environment interactions that contribute to the pathogenesis of CHDs. Implementing a systematic multileveled approach can establish a continuum from phenotypic characterization in the clinic to molecular dissection using combined next-generation sequencing platforms and validation studies in suitable models at the bench. Key elements necessary to advance the field are: first, proper delineation of the phenotypic spectrum of CHDs; second, defining the molecular genotype/phenotype by combining whole-exome sequencing and transcriptome analysis; third, integration of phenotypic, genotypic, and molecular datasets to identify molecular network contributing to CHDs; fourth, generation of relevant disease models and multileveled experimental investigations. In order to achieve all these goals, access to high-quality biological specimens from well-defined patient cohorts is a crucial step. Therefore, establishing a CHD BioCore is an essential infrastructure and a critical step on the path toward precision child health cardiovascular medicine.

## Introduction

Congenital heart defect (CHD) is a structural abnormality of the heart that develops before birth, affecting approximately 1% of live births and is a major source of pediatric morbidity and mortality especially within the first year of life (1–6). The past five decades have witnessed spectacular advancements in diagnostic and surgical interventions in pediatric cardiology (1, 2). However, despite continuing advances in prenatal detection, diagnostic approach, operational procedures, and postoperative care, the CHD field continues to face several challenges.

**Figure 1:**
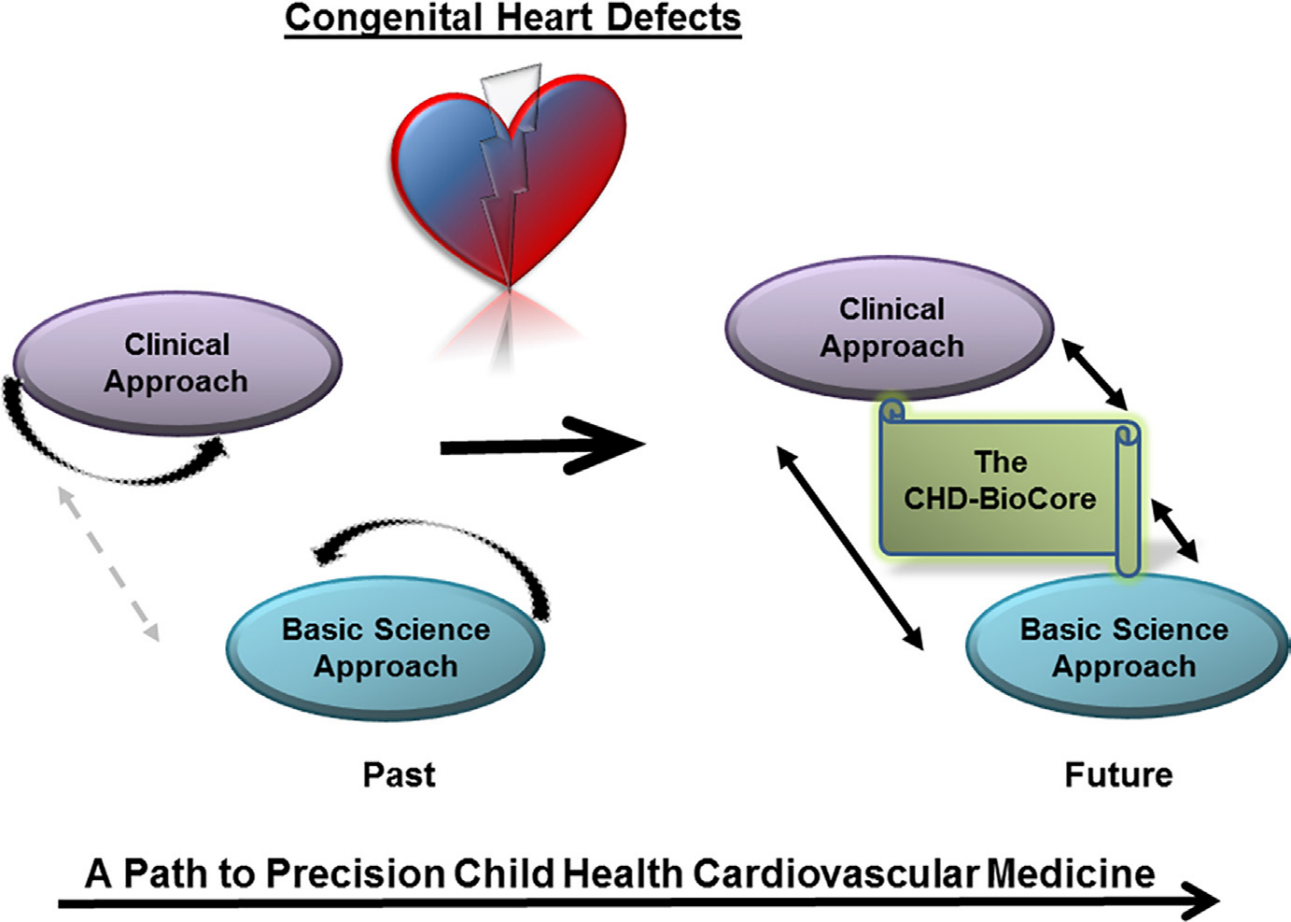
**The congenital heart defect BioCore (CHD-BioCore) implements precision child health cardiovascular medicine**. Illustration of the basic principle of the CHD BioCore to establish an integrated continuum between the bedside and research laboratory with synergized resources and collaborative expertise.

Given the complexity of genetic and environmental factors contributing to the manifestation of CHD throughout the early and late stages (4–13), it is essential to set an effective and practical path to precision medicine for both clinical service and basic discovery. Herein, we propose a multilayered methodology to integrate phenotypic assessment, genetic diagnosis, and analysis of modifying environmental factors in a model system in order to identify targets for prevention of the underlying defect or modification of the disease course. In this review, we describe the elements and use of personalized medicine and systems biology to accomplish this goal. Such integrated approach can accelerate direct translation from discoveries to patient-tailored clinical care and actionable prediction of future outcomes in CHDs (Figure 1).

## Current Challenges of CHDs at Clinical Services

### Early Mortality

Approximately 5% of all newborn deaths were directly attributed to CHDs, with 20% of CHD-related neonatal mortality associated with critical forms of CHDs, defined as CHD phenotypes that require surgery or other immediate procedures to be performed within the first month of life (3, 6). Several clinical challenges may be at the root of this problem. First, significant efforts have addressed the anatomical and hemodynamic aspects of CHDs. However, the morphogenic events that may disrupt cardiogenesis remain partially understood, as are many of the molecular networks that drive normal heart development. Therefore, the mechanistic understanding to the etiology and its link to the phenotypic presentations remain to be further established. Second, how the impaired heart responds to multifactorial stress at birth, including the surgical stress, during early postnatal life is understudied at both the functional as well as the cellular/molecular levels. Third, we continue to have large gaps in our current knowledge on unique features of disease progression in infants vs. adults and right ventricle vs. left ventricle. Importantly, the same underlying genetic/molecular defects can often translate into different severities and therapeutic responses in different individuals. This phenomenon may be due to environmental or other undetected genetic interactions (4–14). Consequently, CHD mechanisms need to be refined at individual and mechanistic levels.

### Lifelong Morbidities

Advanced surgical techniques have led to remarkable success in our ability to palliate or correct modest anatomical defects. As a result, mortality has shifted away from infancy and childhood toward adulthood, with a steady increase of life span for CHD patients (2, 3). As patients get older, acquired morbidities become more frequent due to confounding complications related to the underlying cause of CHD or its treatment. Accordingly, the number of patients with several comorbidities is rapidly increasing, including neurocognitive and social morbidities. In particular, acquired heart diseases, such as coronary artery disease, arrhythmias, cardiomyopathy, and early heart failure, are frequently encountered (2). Hence, our daily clinical encounters nowadays are confronted with a new set of problems (1–3). Therefore, in parallel to technological advancement, there is a need for targeted therapies to optimize life quality and to minimize future complications. In particular, revealing the initial genetic/molecular defect(s) that underlie the CHD, beyond the function and anatomy, is essential to transform our clinical practice to efficient management for those patients and to implement preventive strategies for their families. Future efforts should not aim only to correct or to palliate but also to prevent disease progression and comorbidities. This holistic patient-centered management needs a systematic approach that includes not only skilled surgeons and astute clinicians but also integration with geneticists and molecular biologists with specialized expertise in cardiovascular development, physiology, and pathobiology.

### Phenotypic/Genotypic Heterogeneity of CHDs

During the earliest steps of cardiogenesis, remote developmental signals and local environmental stimuli contribute to the development of a functional circulation and any significant perturbation may produce a maldeveloped heart (7–14). However, dissecting the genetic vs. environmental partition in the pathogenesis of CHDs faces many challenges: first, the phenotypic presentations of CHDs are highly heterogeneous, and so are the underlying etiologies. Second, like many other complex birth defects, the pathogenesis of CHDs has a major genetic origin. However, the recurrence risk within families is minimal with incomplete penetrance and variable expressivity (1–5). Third, genomic studies of CHDs have revealed remarkable genetic complexities (5, 14) including, chromosomal lesions, translocations, gene amplifications, single-nucleotide variants (SNVs), copy number variations (CNVs), and mutations of the key regulatory genes that drive cardiogenesis (4–14). In terms of chromosomal defects, trisomy 21, 13, and 18 cause together around 5–6% of CHD cases with trisomy 21 being the most common (15). Other types of chromosomal imbalance may occur at lower rates including DiGeorge syndrome, a microdeletion of the long arm of chromosome 22 (22q11) (16). Other less recurrent genomic lesions, including a microduplication of the long arm of chromosome 1 (1q21) (17), or the short arm of chromosome 8 (8p23), have been shown to lead to CHD. More recently, a significant genetic association mapped to a 200-kb region on chromosome 20q11 was identified in a genome-wide case–control association study (18). Nevertheless, a child with any of these chromosomal abnormalities may have a normal heart, and there is wide phenotypic variation among those with CHDs. Beyond chromosomal defects, studies of rare Mendelian forms of familial CHDs have revealed numerous monogenic mutations and an expanding list of point mutations of certain genes. For example, mutations of β-myosin heavy chain (MYH7) are associated with increasing atrial septal defects (19). TBX5 and GATA4 mutations produce both atrial and ventricular septal defects (20), and NKX2-5 shows links to septal defects and electrical conduction abnormalities in heart (21). However, mutations in these genes could be found to a lower extent in the more common sporadic form that accounts for 70% of CHD cases.

Although their important roles in cardiac development have been demonstrated (22, 23), the newly emerging contributors to human functional genome, such as splice site mutations, non-coding RNAs, including the long non-coding RNAs (lncRNAs) and the micro RNAs, have been studied far less. Indeed, the lncRNAs and the regulatory elements that reside in the non-coding DNA have recently emerged as important players in the genetic field of cardiac development and defects (4, 24, 25).

### Other Contributing Factors

The evident phenotypic heterogeneity and the variable expressivity, even with the same underlying genetic mutation, indicate that the CHD phenotype is likely contributed by multiple genetic and environmental perturbations. This idea is most supported by an increased risk of CHDs associated with certain environmental alterations during gestation, including maternal illness and medications, folate deficiency, obesity, diabetes, and alcoholism (13, 26, 27). Some of these perturbations may alter cardiac development in a manner that may not be clinically evident at birth, but may result in increased susceptibility to cardiac problems after birth, or increasingly with age. Other than potential toxic and teratogenic effects, these environmental factors may turn on complex epigenetic mechanisms, encompassing DNA methylation, histone modifications (28–32), or the non-coding regulatory elements such as enhancers, promotors, and lncRNAs (4, 24, 25). Identifying such putative factors and mechanisms could provide novel disease modifiers, biomarkers for diagnostics, and targets for prevention.

The cardiovascular health is significantly affected by placental microenvironment. The so-called heart–placenta axis maintains the synchronized development and growth of both organs. However, the factors that drive this intact axis or modify these tightly synergized processes during early embryonic stages remain poorly understood (13). It has been shown that the placental volume and blood flow are major determinants of early cardiac output and fetal organ growth and that placental abnormalities during gestation of the fetus with a CHD have deleterious effects on the outcome of pregnancy, including spontaneous abortion, premature birth, and poor prognosis (33, 34). In a previous study (35), we have shown significant impact on the fetoplacental hemodynamics and fetal cardiac function resulting from intrauterine growth retardation secondary to maternal caloric restriction initiated during the second trimester. Importantly, the reduction of placenta volume and fetal-placental circulatory function was associated with suppression of placental glucose and leucine transport mechanisms.

Coordinated development and function is a highly regulated process during each cardiac cycle and throughout the life span of the individual. Mechanotransduction of blood flow forces in organized 3D networks orchestrates cardiac development to match the functional demands. It has been shown that changes in the retrograde flow during early cardiogenesis, can lead to the progression of cardiac valve defects. Altered hemodynamic status can also influence the development of cardiac cushions, which are valve progenitors (13). The contribution of impaired hemodynamics to the progression of valve and cushion defects has been modeled in Quail embryos with neural crest cell (CNCC) ablation, however, the exact mechanism remains to be established (36).

Literature evidence (37–39) on human and mouse has revealed that folate (folic acid, FA) deficiency, the mood-stabilizing drug lithium (Li), or alcohol exposure can all elevate plasma homocysteine (HCy) and induce cardiac and placental defects. These effects implicate the canonical Wnt/β-catenin signaling and the mediating transcription regulators Hex and Islet-1 in cardiogenic crescent development during early cardiogenesis and their potential role in CHDs. While the outflow tract defects are associated with elevated HCy, maternal Li therapy is associated with Ebstein’s anomaly of the tricuspid valve. Importantly, further evidence suggests that these defects can be prevented by efficient FA supplementation (39). Hence, evaluating the the genetic variations of folate metabolism pathways would likely provide cues for prevention.

More recent evidence has demonstrated that lipid metabolism is also altered after alcohol ingestion in association with folate deficiency and type I diabetes (40). Interestingly, the lipid-related alterations, including fatty acid oxidation, are more predominant in male compared to female embryos, suggesting a gender bias. It is well known that gender influences the clinical presentation and the management of certain acquired cardiovascular diseases, such as coronary artery diseases. Gender differences also exist in the arrhythmogenic risk in patients with inherited or acquired long QT syndromes (41). Likewise, gender differences are also appreciated in CHDs. One such example is that bicuspid aortic valve has a male predominance of 3 to 1 (42, 43). In addition, several population-based studies have shown that more severe types of CHDs are more prevalent in male neonates and those male newborns undergo more severe types of surgeries than the female counterparts (44). Some animal studies also indicate a role for embryonic gender in how genes are expressed. Whether the potential role of gender is dictated by the sex hormones or driven by difference in genetic susceptibility or epigenetic influence between males and females during early embryogenesis requires further studies. In short, more mechanistic investigations are essential to inform CHD management and strategy for prevention.

## Current Progress and Limitations of CHD Research

### Biological Complexity and Gaps of Knowledge

Elegant developmental and genetic studies over the past two decades have revealed the core transcription factors that drive the key transcriptional programs during heart morphogenesis, and demonstrated that the perturbations of these factors result in cardiac malformation, thereby, revealing important triggers underlying CHDs (5–11). Consequently, causative genes were identified, animal models were developed, and new diagnostic and therapeutic modalities were investigated. Collectively, the acquired knowledge has reflected new insights to improve clinical management, which was further empowered by the improvement of physiological monitoring, diagnostic imaging, and surgical applications in CHDs (44–47). However, these approaches mostly focused on observed anatomy, functional indices, or hemodynamic defects, but rarely involved insight at molecular and cellular levels. Further, when the disease under study has heterogeneous phenotypes and complex etiological components, it becomes much more difficult to dissect out the contributing factors and to distinguish between causation and correlation. For instance, just recently, we started to understand why a child has a septal defect, while one of the siblings needs a pacemaker.

The big problem that lies behind every individual CHD patient is to determine what the proper genetic model should be (if any). If analysis from family (familial) or parents (*de novo* recessive) is informative, one can fine-tune the genetic analysis based on the pedigree/familial knowledge. However, for most of the patients, this is not the case. The most likely scenario is that multiple genetic and epigenetic factors interact in any given patient with a CHD, explaining the complex trait character and presentation seen in most cases.

### Systematic Gaps between the Clinic and the Bench

**Figure 2:**
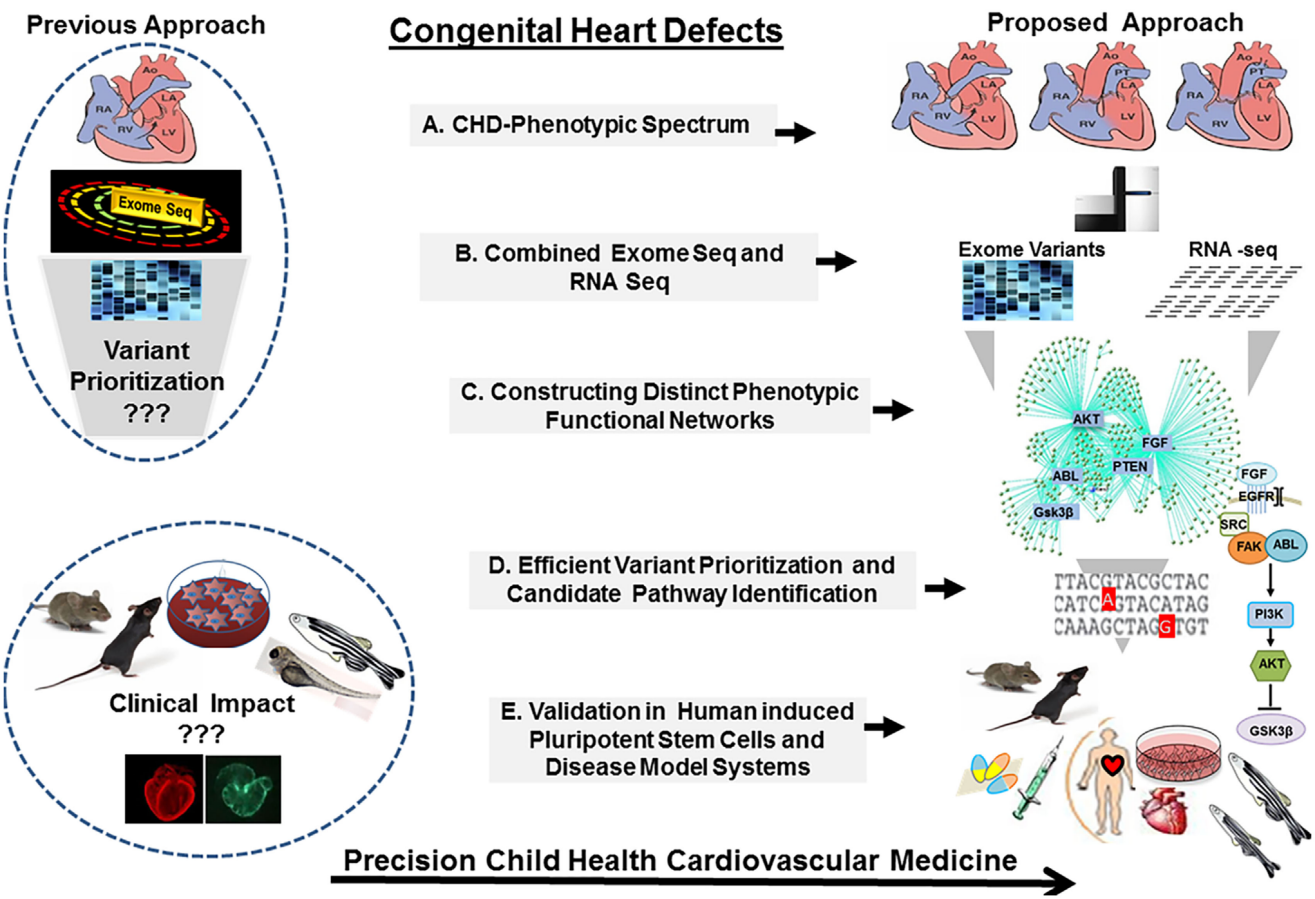
**The congenital heart defect BioCore (CHD-BioCore): strategy and frame work**. Illustration of the strategic framework for the CHD BioCore: (1) Proper delineation of the phenotypic spectrum of CHDs. (2) Defining the molecular genotype/phenotype by combining whole-exome sequencing (WES) and transcriptome analysis. (3) Integration of phenotypic and molecular datasets to identify distinct molecular function networks associated with CHD phenotypes. (4) Integration of genomic data by mapping the putative variants to the molecular networks for mutation prioritization or pathway identification. (5) Mechanistic validation in relevant disease models and human induced pluripotent stem cells (iPSCs) for translational application.

Given such overwhelming complexity, it is a challenge to continue on the trajectory of success in scientific discoveries and clinical advancement unless our approaches to CHDs are operated as a continuum allowing close integration between the clinic and the bench. Moving forward, a new multileveled approach should be implemented. Techniques designed to, specifically, address genetically complex diseases will provide the greatest hope to unravel these clinically important mysteries. Defining CHD cohorts will provide unique opportunities to transform the insight from bedside physiologic understanding and bench side basic science discoveries to risk stratification and patient management based on the causes of the disease. Utilizing molecular phenotyping and targeted genetic screening will allow the identification of deleterious genetic mutations. Developing new strategies to explore the contribution of the environmental and epigenetic modulators and gene modifiers in CHDs and carrying out validation studies in suitable disease models, including patient specific induced pluripotent stem cells (iPSCs) and animal models generated by novel genome editing tools, will allow establishing the causality. Finally, knowledge of the causal determinants will provide a framework to understand CHDs as a continuum from malformation to ventricular failure, from cardiac hypertrophy to arrhythmia, presenting a clear pathomechanistic explanation from the clinical phenotype to therapeutic response, future outcomes and potential models for early prevention (Figure 2).

## Next-Generation Sequencing (NGS) Technology in CHD Field: Advancements and Challenges

Our ability to perform structural and functional genomic studies has been revolutionized with the advent of NGS technologies. The rapidly falling total cost of whole-exome sequencing (WES) and RNA-sequencing (RNA-seq), and in the future whole-genome sequencing (WGS), makes NGS more affordable to approach CHDs both in clinic and at the bench. Nevertheless, along with tremendous advancement, several challenges have arisen with each technique. Therefore, the limitations of each of these individual approaches in detecting novel, potentially disease-causing, variants should be considered.

### Whole-Exome Sequencing

As most of known disease-causing mutations reside within protein-coding regions, the sequencing of the exome (the entire set of all exons in the human genome) has been widely and successfully applied, both for clinical and investigative studies (48–56). WES has been employed for the identification of heritable germline mutations underlying Mendelian disorders (51), somatic mutations in cardiomyopathies, *de novo* mutations in CHDs, and other complex birth defects. Compared to SNP (single-nucleotide polymorphism) microarray, WES has higher sensitivity and specificity for detecting single-nucleotide variants than small insertions and deletions (indels). In addition, by using the depth of coverage from mapped short-sequence reads and appropriate bioinformatics tools (54), WES can detect copy number variations (CNVs), but it is not the method of choice. These properties make WES more robust to study complex disorders with genetic and phenotypic heterogeneity such as CHDs, even for rare and sporadic cases. However, moving beyond the coding regions, research on CHDs will benefit tremendously from replacing WES eventually with WGS (53–55), particularly, for the detection of pathologic CNVs and altered functional non-coding elements, when the sequencing cost of the whole genome becomes more affordable in the near future, using better algorithms of mapping the non-coding region and longer sequence reads generated by NGS technologies.

In terms of cost, applying WES studies is now more affordable for application in CHDs diagnostics and research studies, with several advantages over the laborious conventional targeted sequencing of candidate genes, in particular when performed in trios. However, compared with WGS, additional exome-enrichment steps are required for WES, adding to the overall cost. In addition, the incomplete capture of some exons and variable sequencing depth could potentially lead to false-negative results and difficulties in detecting CNVs. In terms of sequencing depth, similar to WGS, an average sequencing depth of 30–50× for WES of genomic DNA is usually sufficient to detect most germline SNVs accurately (51, 52). However, greater sequencing depth would be required to detect somatic point mutations in order to account for tissue contamination and genetic heterogeneity within the tissue. Further, the use of WES as a diagnostic tool continues to face technical hurdles and ethical issues involved in adopting this approach in clinical practice for CHDs. Specifically, analyzing the large amount of sequencing data that typically generates >30,000 genetic variants per exome is time and effort consuming. Thus, a robust variant-filtering pipeline must be generated to identify putative disease-causing variants for CHDs population or a particular subcohort. After completing several bioinformatics and filtering steps including identifying the non-synonymous coding variants, one approach is to measure the average frequency of “new variants” per gene across the genome, as determined by comparing the newly sequenced exomes to the common variation found in dbSNP. Challenges to such approach will arise as the finding is generalized to other CHD subpopulations or phenotypes. In addition, as the number of sequenced exomes rises, dbSNP will carry overwhelming number of uncommon variants. At present, new population-based databases are available such as the Genome Aggregation Database, also known as the Exome Aggregation Consortium in its first phase, which provide the first large-scale public exome CNV variant dataset based on ~60,000 human exomes exclusively (54, 55). More comprehensive lists of these and other recommended databases are available from the American College of Medical Genetics and Genomics (ACMG).

New statistical metrics are required to distinguish false positives from true signals and to account for locus and etiologic heterogeneity, which may often be underrecognized. In terms of the accuracy of variant detection, the NGS platforms generally have higher raw base reading error rates than traditional Sanger methods. Therefore, although WES technologies have greater specificity to detect both germline and somatic SNVs, further validation using Sanger sequencing remains a common practice. Finally, considerable ethical concerns (56) and debates remain unresolved, including interpretation, reporting, and disclosure of findings at standard level of diagnostic test accuracy.

### Whole-Genome Sequencing

At present, WGS is the most comprehensive tool for genome-wide variant identification, ranging from SNVs and small indels to larger structural rearrangements, covering the coding sequences as well as the non-coding regions. Compared to WES, implementing WGS brings important additional advantages to the CHD field. Other than uncovering potentially functional lncRNAs and important gene regulatory elements such as promoters and enhancers, CNVs could be readily detected by WGS, thereby, overcoming notoriously difficult limitations of WES (57–59).

The CNV is defined as a stretch of DNA, longer than 1 kb, which presents in the genome with an abnormal number of copies. The CNVs can constitute large deletions and duplications, as well as unbalanced translocations (54). In WGS data, large deletions can be easily detected by the same paired-end methods for detecting moderately sized deletions. However, large duplications are more difficult to detect, as there is no single read or read pair spanning the insertion. Detecting large insertions could be achieved using one of the split-read methods such as Pindel, which uses a pattern-growth approach to detect breakpoints at which the sequenced genome diverges from the reference. These methods, including discordant paired-end and split-read, generally do not perform well for CNV detection from WES data or targeted-capture sequence data due to the increased GC bias inherent to targeted-capture data and the discontinuous nature of exome sequencing as well as the difficulties in localizing the breakpoints. In this case, algorithms that examine the sequence depth of coverage are the primary means for detecting CNVs. Several software packages have been developed to address these issues (54, 55). However, because of the small size of targets in typical exome-capture data, many current algorithms for CNV detection require either a paired normal sample or a panel of population controls. Further improvements of these methods are required.

Together, WGS is the ultimate approach for genetic testing in CHD precision medicine. However, it will still take much more efforts before it becomes a standard practice in clinical setting. Many challenges are yet to be resolved including large sample size requirement, financial cost, and current limitation in interpretation of variants detected in non-coding regions. In the meanwhile, it should be recognized that WES provides an essential stepping stone toward implementing WGS. Implementing and refining the knowledge acquired from population databases for both pathogenic and benign CNVs identified in WES and WGS data will be crucial for developing effective strategies to interpret clinically relevant variants. Moving forward, analyzing database generated from the 1000 Genome project will provide new insights into the nature and extent of human genetic variation, leading to optimized variants detection, filtering, and prioritization (59).

### Incidental and Secondary Findings from WES/WGS

Variants may be detected during the course of clinical WES/WGS that are not directly relevant to the primary clinical indications of the patient, so-called “*incidental*” findings. To address such variants, the ACMG has provided guidelines (60–62), which initially recommended that clinical laboratories report any known or suspected pathogenic variants detected in any one of a list of 56 genes regardless of patient age. However, these guidelines have since been revised (62) to allow patients to opt out of receiving these types of results, and the term *incidental findings* has been formally replaced by *secondary findings*, which reflects a desire for laboratories to actively search for these types of variants during the process of clinical testing. While some laboratories have chosen to adhere closely to the guidelines, other clinical laboratories have chosen to keep these variants as true incidental findings. Therefore, the consent for genomic analysis should be explicit, with reference made in the consent process to the possibility of incidental findings. It is also important that both the patients and the ordering physicians understand the laboratory specific approach to incidental/secondary variant reporting and are aware of the possibility that incidental/secondary findings will appear on WES/WGS report if a patient has opted and indicated to receive them on the informed consent form provided by the clinical laboratory.

Most of the recommended 56 genes involve autosomal dominant cancer predisposition syndromes, one such example being *BRCA1* that predisposes an individual to breast cancer, when a referral to an oncologist may be necessary.

For obvious reasons, however, handling the issue of incidental/secondary findings in children is far more complex and requires careful considerations of more sensitive issues (60–62). Important concerns have been raised regarding. (1) The potential of emotional harm of labeling a child as being at risk to develop a condition years into the future. (2) The potential medical benefits to the child’s parents of receiving certain information (e.g., *BRCA1* and cancer predispositions). (3) The need to preserve the child’s autonomy and rights regarding her/his future ability not to have information about their own genetic constitution. One approach to address these issues is to accept the disclosure of the minimum package of important information (the list of 56 genes) as per the ACMG recommendations but no other information until the subject can make his/her own decisions as a competent adult. Nevertheless, consensus has yet to be made for more complex scenarios, such as genetic testing of a fetus, newborn screening, later-onset neurodegenerative diseases, psychiatric disorders, and cardiology disorders associated with sudden death susceptibility.

### RNA-seq for Molecular Phenotyping

As with WES and WGS, RNA-seq is a genome-wide approach and a powerful information-generating NGS tool. Hence, it would be very applicable to diseases characterized by genetic and phenotypic heterogeneity such as CHDs (63, 64). The transcriptome of a given tissue represents a complete set of the transcribed genome and encompasses all the transcripts, including the protein-coding mRNAs and the non-coding RNAs such as miRNAs and lncRNAs (65). Further, by illustrating the quantity and expression patterns of thousands of genes simultaneously, RNA-seq provides a dramatic increase in our knowledge of the transcriptional landscape and regulation, including insight into functional pathways and regulation of biological processes of coordinated genes during development, under different conditions and in different disease phenotypes. Furthermore, RNA-seq allows determination of the transcriptional structure of genes, in terms of their transcription start sites, splicing patterns and other posttranscriptional modifications (66), in addition to allele-specific expression in CHD (67) and fusion genes secondary to chromosomal rearrangements (68). Moreover, several RNA-seq methods have been developed to specifically sequence the 3′ end region of transcripts, allowing the discovery of alternative polyadenylation sites (69). Likewise, several RNA-seq analysis methods of reads covering exon–exon junction have facilitated examining alternative splicing events (65). Finally, RNA-seq also allows detection of SNPs in transcripts by comparing them to a reference transcriptome and quantifying the modification in expression levels of each transcript in affected population compared to control. A particular advantage of such application is allowing the discovery of SNPs in the non-coding sequences, including the lncRNAs (63, 64). Nevertheless, relative advantages and limitations also exist in applying transcriptome studies solely in the context of variant detection. In particular, the challenge of detecting mutations in completely untranscribed genes (null mutation) using this approach must be appreciated. Further, the transcriptome is tissue/cell specific and may vary with time; hence, the transcriptome of a specific tissue only partially reflects the exome (70). Another critical challenge to the detection of variants using RNA-seq is the considerable variability in transcript expression levels, which can be mitigated by increasing the sequencing depth. However, the sequencing depth or coverage of RNA-seq is difficult to be estimated, given the potential coverage of different isoforms for the same gene in addition to variation in transcript abundance in different tissue, factors that may lead to considerable noise. Further, the optimal number of biological replicates (71, 72) that would be needed to ensure a valid biological interpretation of the results remains unclear. Finally, RNA-seq technologies continue to challenge bioinformatics for the large amount of multilayered data.

### Other Omics Platforms

The molecular characterization of a CHD phenotype could be furthered and enriched by adding other functional information derived from utilizing other omics platforms, when available, including the epigenome, the metabolome, and the proteome.

## Implementing Precision Medicine in CHD: The Congenital Heart Defect-Biocore (CHD BioCore)

**Figure 3:**
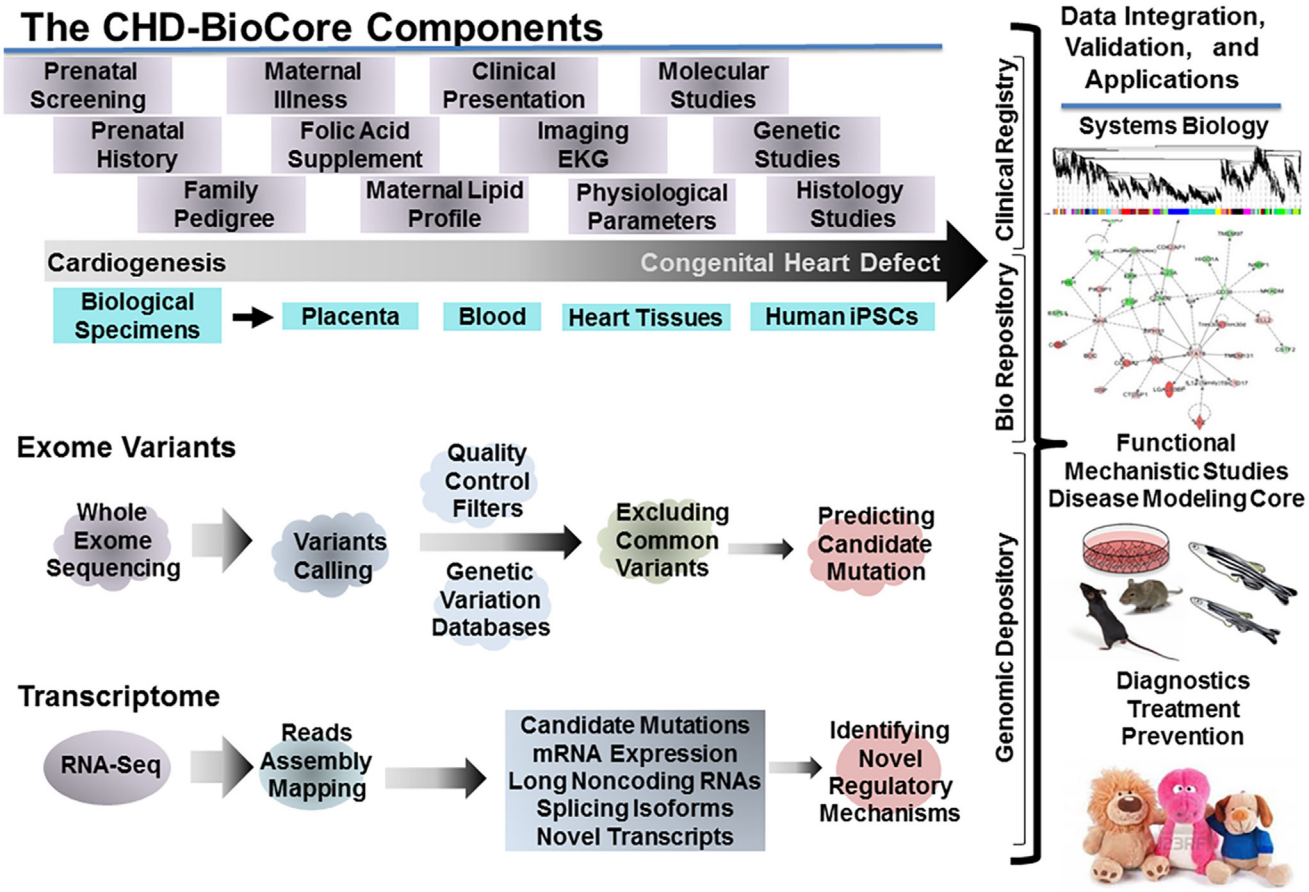
**The congenital heart defect BioCore (CHD-BioCore): organization and network**. Illustration of the central components of an impactful CHD BioCore for integrated multisystem approach: (1) The Clinical Registry. (2) The Bio Banking Repository. (3) The Genomic Depository. The key elements and resources for each component as well as the integration among them by implementing systems biology approach were also illustrated.

For implementing a path to precision child health cardiovascular medicine, it is important to stratify the phenotypic spectrum of each CHD into subcohorts, based on structural, physiological, and functional parameters, delineate the distinct genotype associated with each subphenotype (subcohort) utilizing the power of NGS (WES, WGS), establish the pathogenic molecular signature that discriminates the extreme ends of each subcohort by implementing RNA-seq and other omics data when available (methylome, proteome, and metabolome). The integration among these key resources by employing systems biology tools is an auxiliary step. An important infrastructure to achieve these steps requires creation of a continuum from the clinical presentation, hemodynamic indices, and physiological parameters, to genomic determinants and gene expression landscape. Therefore, direct access to biological specimens is indispensable (Figure 3).

### The Congenital Heart Defect BioCore (CHD BioCore)

Availability and quality of heart tissue specimens from pediatric patients with CHDs with well-defined phenotypes is the rate-limiting factor in any effort to achieve clinically relevant scientific advances in the CHD field. Hence, establishing a CHD BioCore is an invaluable infrastructure to accelerate scientific discovery and clinical translation by synergizing expertise from a collaborative team from different departments with combined clinical and research expertise. The BioCore also serves to interact with other CHD network member institutes nationwide and with the Pediatric Cardiac Genomics Consortium.

#### The CHD BioCore Components

An impactful CHD BioCore should have three central and complementary components: (1) CHD Clinical Data Registry. (2) CHD Bio Banking Repository. (3) CHD Genomic Data Depository.

##### CHD Clinical Data Registry

Defining CHDs cohort and clinical pool is the first priority for achieving comprehensive delineation of CHD phenotypes. Simple and cost free enrollment process and open eligibility criteria need to be utilized to achieve large-scale CHD population without any restriction on ethnic backgrounds, sex, or age. All efforts should be made to identify familial cases and enroll other siblings and extended family members, including healthy and affected individuals. Printed materials and simple pamphlets (brochures) containing descriptive or advertising material can be utilized to facilitate recruitment.

Clinical data collection should be initiated through subject and family interviews in conjunction with retrieving medical record information including hospital course, surgical procedures, and medical interventions. The data should be stored on structured electronic forms in the CHD clinical data registry. All data should be individually coded before storage. The study code will allow linking the data to the corresponding subject within the CHD BioCore cohort. Pertinent clinical data registry collection includes (1) Demographic data. (2) Detailed cardiac, medical, surgical, and developmental history. (3) Review of systems including dictating presence of associated syndromic features. (4) Clinical genetic tests and results. (5) Detailed prenatal course, prenatal genetic screening, fetal physiologic profile, hemodynamic parameters, and imaging results. (6) Detailed maternal obstetric history including, maternal medical illness, and potential therapeutic and environmental exposure, such as Li ingestion, maternal diet, the status of folate supplement, and lipid profile. (7) Detailed family history and three-generation family pedigree. (8) Cardiovascular imaging data that may allow precise delineation of CHDs anatomy. (9) Denominated disease course, perioperative parameters, subsequent complications, and associated morbidities, such as developing coronary artery disease, metabolic syndrome, and neurological and cognitive disorders that is essential for future disease prediction. (10) Follow-up questionnaire that investigates cardiac and non-cardiac medical and surgical events, medication usage, and neurodevelopmental status. (11) Finally, in addition to affected patients (index cases), the BioCore enrollment and the data collection process should include healthy twins, siblings, extended family members, and control volunteers. The clinical registry data should be reevaluated periodically in view of the newly acquired research discoveries to explore potentials for translation and to improve outcomes. A study coordinator with specific training in large data management may assist in patient enrollment in a genetic study and ensure the data completion and accuracy.

##### CHD Bio Banking Repository

Proper collection, coding, and bio banking of the biological specimens in a timely, sensitive manner is imperative to achieve precise molecular and cellular signature. Proper procurement and annotation of remnant human tissue samples provides critical resources for gene discovery and translational research. Rapid and coordinated processing and storage of biological specimens can allow presenting a snapshot of protein and gene expression, genetic and epigenetic alterations, protein and enzyme activity, and/or cellular and tissue architecture through collecting: (1) Specimens from cardiac tissue serve to perform molecular cellular phenotyping, transcriptomic, and proteomic studies. (2) Specimens from fetal/embryonic cardiac tissue obtained from abortion and fetal demise cases. (3) Blood/urine/saliva samples from index subjects and from parents and affected family members to obtain genomic DNA for molecular genetics and genomic studies including WES/WGS. (4) Specimens from the placenta and the cord tissues, and cord blood samples. (5) Skin biopsy and blood samples collected from index cases and parents to generate human iPSCs. (6) Disease-specific human iPSCs derived from white blood cells to be utilized for mechanistic validation and testing new therapies. (7) Control samples. A potential resource for obtaining control specimens is the heart transplant donors and other healthy donors who died because of a non-cardiac reason. However, this resource may be challenging with obvious difficulties including the complexity of the informed consent process. Therefore, deriving the hiPSCs from the white blood cells or the skin fibroblasts from affected individuals as well as healthy family members and other control participants is an essential and efficient solution.

##### CHD Genomic Data Depository

Achieving molecular characterization of CHD genotypes is essential to study CHDs mechanism. The CHD genomic data depositary is an essential component to build and enrich the CHD BioCore. The raw datasets generated from WES/WGS/RNA-seq and other omics studies of CHDs cohort should be deposited in the CHD Genomic Data Depository to facilitate comparative analysis, network construction, and integration with clinical and experimental data as we have detailed in Section “The CHD BioCore Operation.”

#### The CHD BioCore Operation

In order to dissect the underlying complexity and biological heterogeneity of CHDs, integrating the phenotypic data, the molecular signature, and the genomic background is both essential and challenging. In particular, extracting biologically relevant targets for clinical intervention and gene discovery is critical.

##### Integrated Genomic Approach (WES and RNA-seq)

As with other large-scale sequencing projects for complex diseases, a key challenge in large-scale sequencing approach for CHDs is to distinguish putative “damaging” mutations, which may contribute to malformation, from “non-damaging” mutations, which are functionally neutral. The second key challenge is to identify novel developmental pathways that may be perturbed by uterus environment during heart development, leading to cardiac defect by affecting cell proliferation, apoptosis, migration, angiogenesis, etc. The third and particularly critical challenge is to transition from association and correlation to function and causality. Although conflicting, a number of approaches have addressed these challenges in other diseases, as well as in CHD pathobiology, attaining some degree of success (73, 74). However, several analytical challenges of large data rapidly emerged, requiring new tools to integrate genomic information with traditional clinical parameters, environmental culprits, and pathological data in an iterative manner.

Our limited ability to interpret the functional significance of genetic variants requires moving from the change in gene sequence to the functional impact of that change, therein, revealing critical needs for interrogating both WES (WGS in the near future) and RNA-seq data (Figure 2). Such bidimensional genomic approach has provided efficiency in several models of complex disease including autism spectrum genetics, cancer, and long QT syndrome therapies (75–77). In particular, combining exome and transcriptome data has been essential for the definition of fusion genes, allelic heterogeneity, locus heterogeneity and splice site mutations (66, 67). Other than enhancing data interpretation, candidate variant selection and diagnostic precision, the inherent output of RNA-seq, including defining the contents and the abundance of protein-coding genes, splicing isoforms, and non-coding RNAs, could be utilized to a larger extent to study CHDs mechanisms, such as the non-coding elements and genetic modifiers.

##### Systems Biology Methods for Network-Oriented Data Mining

A comprehensive approach for CHDs requires sophisticated management and integration of giant datasets, including genomic, expression, cellular and molecular, environmental, phenotypic, and clinical datasets. Systems biology methodology is rapidly developing and particularly intriguing solution for constructing, evaluating, and interpreting gene networks from large datasets, to predict the biological impact of novel signaling pathways and to prioritize deleterious candidate mutations (78). In the current review, we take a wide definition of systems biology by considering a CHD in a patient as a system. In this context, systems biology approach is the study of the integrated interactions of the network(s) of genes, their variants (polymorphisms, rearrangements, alternate splicing, and mutations), their isoforms, and the molecules with which they interact to execute the signaling cascade or the biochemical reactions that reflect the function of that system that ultimately leads to the specific phenotype.

##### Data Integration Algorithm

To establish an algorithm for data integration, we propose *de novo* construction of functional networks based upon the RNA-seq data, and other omics data when available, by applying weighted gene network co-expression analysis methods (WGNCA) (78), so as to build both combined and phenotype specific co-expression networks. This approach assumes that genes with similar expression patterns across different samples share similar regulatory program and may function in common or similar biological pathways or cellular processes. In contrast the genes with different expression patterns across different samples may reflect differential pathway activation or altered regulation of cellular processes that are specific or unique to each sample. First, the molecular signature that defines a CHD phenotype, or a subcohort, needs to be characterized separately by interrogating the different layers of the transcriptome complexity derived from RNA-seq and constructing comprehensive biological networks from the coding mRNA isoforms and the non-coding RNA transcripts. Next, by implanting correlation statistics using a composite measure of module gene expression, also known as the module eigengene, or the first principal component, the significance of association between a specific module and a trait, such as a clinical phenotype, an environmental exposure, or a genetic mutation, could be calculated. This will serve to link the molecular module to the trait of interest. The impact of incidental confounding covariant could be adjusted using the regression statistics. In this manner, the large datasets for each clinical phenotype can be reduced into small groups of co-expressed genes, known as gene modules. Each module represents a unique and distinct co-expressed gene network.

Then, a preservation analysis serves to compare the modules that are common and those that define the traits of interest, a clinical phenotype, an environmental exposure, or a genetic mutation. Using the spectrum of *Teratology of Fallot* as an example, the cases could be stratified clinically along a spectrum of severity based on their anatomical structure, such as the degree of pulmonary stenosis as the *Z* score. After constructing the modules that associate with each case (or a group of cases with similar degree of stenosis), the preservation statistics, e.g., hypergeometric overlap, can be used to determine the module(s) that are common vs. those that define each end of the spectrum (the mild, the moderate, or the severe subphenotype), thereby, the putative functional molecular networks overlapping along the phenotypic spectrum and those that define each subphenotype will be constructed. The poorly preserved modules in each analysis may represent a distinct molecular signature of each subphenotype. Further, the corresponding hub genes, those top significant genes that highly correlate with the module eigengene, may represent a primary indicator of the network function for the subphenotype. Finally, the biological function of the unique hub genes or distinct network could be predicted using functional ontology and pathway enrichment analysis methods using DAVID.

In parallel analysis, the genes affected with putative deleterious variants obtained from WES analysis should be examined for their biological function. Next, the candidate variants will be mapped to the constructed molecular networks of each subphenotpe. Subsequently, the highly affected genes at the expression level or posttranscriptional modification can be identified, allowing to link to a distinct expression module, which correspond to a defined subphenotype, to a specific mutation of a key transcription regulator or a hub pathway that bears an excess mutation burden of several genes.

An important advantage of this systems based strategy is the applicability to construct functional networks from multiple data sources, including splicing isoforms, non-coding RNAs, or proteomic, metabolomic, and epigenetic data, when available, as well as other structural, functional, clinical, and environmental parameters in functional modules. Therefore, this strategy presents a potential tool for determining the functional regulatory network, based on which, the variants selection and prioritization will be carried out by interrogating the DNA data obtained from WES/WGS to be validated in mechanistic studies. In this scenario, this precise phenotypic/genotypic characterization at the molecular functional level will tremendously empower pinpointing and prioritization of the candidate damaging variants for mechanistic studies and future translation to diagnostic applications and targeted therapy.

In a recent study (24), we demonstrated the utility of systems biology for interrogating the coding and non-coding transcripts derived from RNA-seq data to detect functionally important (even novel unannotated) lncRNAs potentially implicated in cardiac maturation and CHDs phenotypes. These lncRNAs potentially interact with regulatory elements or epigenetic regulators to produce significant biological alterations and protein damages. Comprehensive systems biology models have been employed in autism spectrum disorders (55) and cancer biology (79) for integrating multilayered clinical data and genomics networks. Other intriguing systems based methods include the Integrated Personal Omics Profile (IPOP) for personal omics for dynamic data integration along the clinical course (80), allowing acquiring a comprehensive picture of detailed molecular differences between different physiological states as well as during the pathogenic course from onset to resolution.

Another method starts by identifying the core pathways that appear to be implicated in CHDs based on existing knowledge, and constructing gene–gene interactions pathways to merge them into a new expression network and then, mapping all mutated genes to the newly constructed network. The shortest paths connecting all mutated genes could be filtered from the network to form a subnetwork. Subsequently, the network modules could be analyzed to identify candidate pathways for mechanistic studies. A similar approach has implicated the ciliopathy pathway in CHDs (81) by using WES and constructing protein–protein interaction networks.

Lastly, in light of the clonal nature of cell expansion in cardiovascular development (7) and given the majority of CHD cases occur on a sporadic basis with the heart being the only affected organ, somatic mutation may also have a significant contribution to CHDs. Therefore, constructing mutual exclusivity modules (MEMo) (82) may serve to predict novel hub gene candidates from a specified gene network involving somatic mutations and to construct the interaction network in CHDs without prior information of the underlying gene–gene interactions. This method is based on the “Mutual Exclusivity Principle” which implies that different gene mutations or alterations that affect the same pathway do not tend to co-occur in the same patient. In this method, a gene exclusivity network can be constructed based on the exclusivity score, which acts as the weight of edge between every pair of genes. The MEMo are then extracted and linked to RNA-seq modules using specific statistical algorithms.

##### Integration of CHD BioCore in CHD Clinical Care and Research

As previously stated, the CHD BioCore infrastructure should be organized to synergize expertise from a collaborative team with combined clinical and research expertise. Specifically, it needs to build seamless interactions with multi-omics platforms, where WES and whole transcriptome profiles can be accomplished and future proteomics or metabolomics can be implemented. The discoveries made through systems studies of CHD samples will need to be validated and mechanistically interrogated in model organisms. Mutagenesis and animal model studies remain needed to lend further support to the causality of the variants and to demonstrate the effect that causal variants have at phenotypic level. Model systems for CHD gene discovery may include (1) Cultured cells, including primary cardiomyocytes, endothelial cells, and fibroblasts from developing rat or mouse hearts and hiPSCs derived from healthy controls and CHD patients, which may offer mechanistic clues at molecular and cellular levels. (2) Zebrafish models combined with genome editing via CRISPR/Cas9 system, which offers high efficiency *in vivo* analysis for cardiovascular development. (3) Target-specific genetically engineered mouse models that offer in-depth analysis for functional, pathological, and translational validation. Such model systems can then be validated using appropriate pathologic, biochemical, or physiologic assessments (Figure 3).

#### The CHD BioCore Data Sharing

As the path toward understanding CHDs progresses, knowledge sharing through informatics databases will accelerate the dissemination and validation of experimental findings.

## Conclusion

An effective clinical utilization of the new discoveries promises to guide strategic approaches toward improved future outcomes for CHDs but requires narrowing existing gaps between the laboratory bench and the clinic. The importance of dissecting the molecular mechanism of CHDs is at least threefolds. First, linking CHDs with specific genetic mutations, epigenetic or molecular markers, or environmental exposure can lead to disease prevention, family planning, and possible intervention prior to birth. Second, better knowledge of the genetic and molecular basis of CHDs would allow more mechanism-based, precise, and personalized approaches to these patients in the prenatal and preoperative setting. Third, with better understanding of gene–gene interaction and gene–environment interaction involved in CHDs, we will be able to identify novel targets and intervention approaches to prevent and effectively treat the pathological manifestation of CHDs. Finally, establishing CHD BioCore is essential infrastructure serving to integrate clinical efforts and research innovation. We thus describe a procedure to, effectively, reduce observations to a focused subset of high specificity on the path to precision child health cardiovascular medicine.

## Author Contributions

Conception, design, and drafting of the manuscript: MT. Substantial contributions to the conception: BR, SN, and YW. Substantial contribution to the design of the work, revising the work critically, final approval of the version to be published, and agreement to be accountable for all aspects of the work in ensuing that questions related to accuracy or integrity of any part of the work are appropriately investigated and resolved: MT, BR, NH, JA, JF, SN, and YW.

## Conflict of Interest Statement

The authors declare that the research was conducted in the absence of any commercial or financial relationships that could be construed as a potential conflict of interest.

## Abbreviations

CHD, congenital heart defects; lncRNA, long non-coding RNA; NGS, next-generation sequencing; WES, whole-exome sequencing; WGS, whole-genome sequencing; SNV, single-nucleotide variants; SNP, single nucleotide polymorphism; CNV, copy number variations; MEMo, mutual exclusivity modules; WGCNA, weighted gene co-expression network analysis.
